# Calculating the peak skin dose resulting from fluoroscopically‐guided interventions. Part II: Case studies†

**DOI:** 10.1120/jacmp.v13i1.3693

**Published:** 2012-01-05

**Authors:** A. Kyle Jones, Alexander S. Pasciak

**Affiliations:** ^1^ Department of Imaging Physics, Division of Diagnostic Imaging The University of Texas MD Anderson Cancer Center Houston TX 77030; ^2^ Department of Radiology University of Tennessee Medical Center Knoxville TN 37920 USA

**Keywords:** peak skin dose, fluoroscopy, dose report, radiation injury, dose

## Abstract

Part II of this review article presents four case studies for which peak skin doses are calculated following the methods outlined in Part I of this review. The data available in the cases ranges from proprietary dose reports to fluoroscopy time and number of digital acquisition frames only. Flowcharts are provided for each case. These flowcharts outline the calculation steps and data sources used to estimate the peak skin dose. The accuracy that can be achieved using these methods depends on several factors, including the calibration accuracy of dosimetric equipment, accuracy of information reported in the DICOM header and proprietary dose reports, accuracy of quantities measured by the medical physicist, and procedural factors such as rotation of the C‐arm during a fluoroscopically‐guided procedure.

PACS numbers: 87.59.Dj, 87.53.Bn

## I. INTRODUCTION

In Part II of this work, we present a series of case studies for which peak skin dose (PSD, Note: abbreviations for this term and others used in this manuscript are listed in Table 1) reconstructions have been performed following the methods outlined in Part I of this review.^(^ ^1^ ^)^ Flowcharts are provided to accompany each case. Sources and values for the quantities used are listed in the respective flowcharts. The reader will need to refer to Part I to consult some of the tables and figures referenced in this article. Other authors have in the past used DAP^(^ ^2^ ^)^ or measured X‐ray tube outputs^(^ ^3^ ^)^ to estimate skin doses resulting from fluoroscopically‐guided procedures according to methods they have developed.

**Table 1:** Abbreviations used in this manuscript.

*Term*	*Abbreviation*	*Definition*
Peak skin dose	PSD	The highest dose to a single area of the skin, units of Gy
Fluoroscopically‐guided intervention	FGI	Any medical intervention using fluoroscopy for guidance
Reference point air kerma	$K_{a,r}$	Cumulative air kerma at the interventional reference point, units of Gy
Kerma area product	KAP	Product of air kerma and x‐ray field size, units of Gy‐cm^2^
Digital Imaging and Communications in Medicine	DICOM	Standard for communication used by medical imaging equipment
Interventional reference point	IRP	Point located 15 cm back towards the focal spot from the isocenter of a C‐arm fluoroscope^(^ ^4^ ^)^
Source‐to‐image distance	SID	Distance from the focal spot to the center of the image receptor, units of mm
Field of view	FOV	Size of the x‐ray field at the image receptor, units of cm
Entrance skin air kerma	ESAK	Air kerma measured at the entrance surface of the patient, units of Gy
Source‐to‐patient distance	SPD	Distance from the focal spot to the entrance surface of the patient, units of mm
Backscatter factor	BSF	Factor that is applied to calculate entrance surface dose from the ESAK, accounts for the fact that many X Rays of diagnostic energy are backscattered in tissue, unitless
f‐factor	f	Factor used to convert exposure to dose in tissue, units of Gy/R. A similar, unitless quantity can be used to convert from air kerma to dose in tissue.
Digital acquisition series	DAS	A series of images generated using digital acquisition imaging
Fluoroscopic reference point air kerma	$K_{a,r}(f)$	Total reference point air kerma resulting from fluoroscopic imaging, units of Gy
Digital acquisition reference point air kerma	$K_{a,r}(d)$	Total reference point air kerma resulting from digital acquisition imaging, units of Gy
Total reference point air kerma	$K_{a,r}(t)$	Total reference point air kerma resulting from the sum of fluoroscopic and digital acquisition imaging, units of Gy
Projected x‐ray field size on the skin	$A_{skin}$	The area of the projected x‐ray field at the entrance surface of the patient, units of cm^2^

## II. METHODS

### A. Case 1

A 69‐year‐old man weighing 108 kg underwent spinal artery embolization prior to resection of metastatic renal cell carcinoma. Upon completion of the procedure, a radiologist noted that the $K_{a,r}$ was unusually high and contacted the medical physicist. The physicist immediately printed the proprietary dose report from the fluoroscope. The dose report and information from the DICOM headers of the digital acquisition series were used to estimate the PSD delivered to the patient. A C‐arm fluoroscope with a flat‐panel image receptor was used to perform the procedure. This particular piece of equipment reported $K_{a,r}$, KAP, and a proprietary dose report. Archived images were also available for consultation. This case falls under Scenario 1 from Part I of this review,^(^ ^1^ ^)^ with DICOM information and proprietary dose reports available.

**Figure 1:**
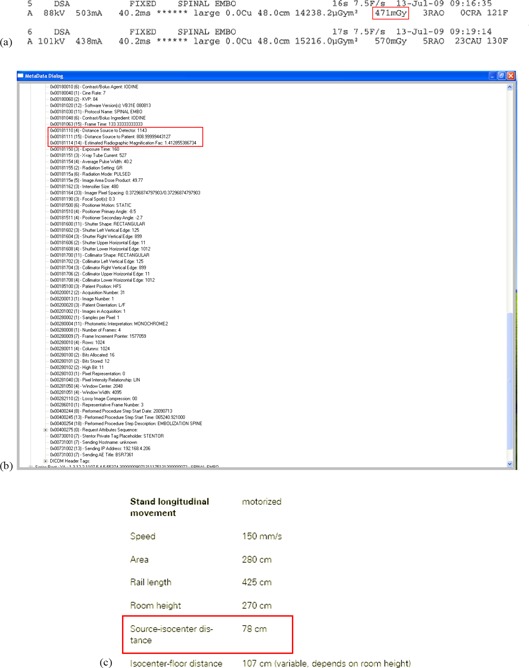
Sources of information used to calculate PSD in Case 1: (a) $K_{a,r}(d)$ for each DAS was listed in a proprietary dose report; (b) distances were listed in the DICOM header; (c) the location of the IRP was listed in the equipment operator's manual.

The calculation was performed piecemeal, with each DAS considered individually. First, the ESAK for each DAS was calculated by applying the inverse square law correction to the reported $K_{a,r}(d)$ for each DAS in the dose report (Fig. 1(a)). The relevant distances were extracted from the DICOM header for each DAS (Fig. 1(b)) and from the equipment manual (Fig. 1(c)). In this case, the IRP (the location of which was given in the equipment manual) and the source‐to‐patient distance, found in the DICOM header, were used. On this imaging system, the source‐to‐patient distance was the actual source‐to‐table distance.

**Figure 2:**
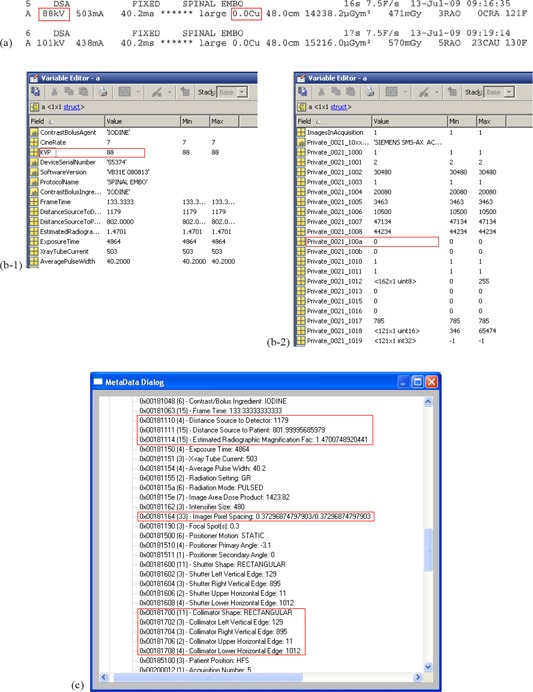
Data used to estimate the BSF and f‐factor. kV and added filtration* were listed in both the (a) proprietary dose report and (b) the DICOM header. Data used to calculate the x‐ray field size (c) were available in the DICOM header. *Cu filter thickness is in tag Private_0021_100a.

Next, the BSF for each DAS was calculated using values for k V, X‐ray field size at the patient location, and added filtration. The kV and added filtration were available in both the dose report (Fig. 2(a)) and the DICOM header (Fig. 2(b)), and the X‐ray field size at the patient location was estimated from the collimator positions, source‐to‐image distance, and source‐to‐patient distance, which were all extracted from the DICOM header (Fig. 2(c)). Application of the BSF and the table and table pad correction to the ESAK yielded the ESD.

The third step was the conversion from ESD to absorbed dose to the skin. The appropriate correction factor was derived from the f‐factor relating dose to skin to dose to air. The application of the f‐factor to the ESD yielded the skin dose for each DAS.

**Figure 3:**
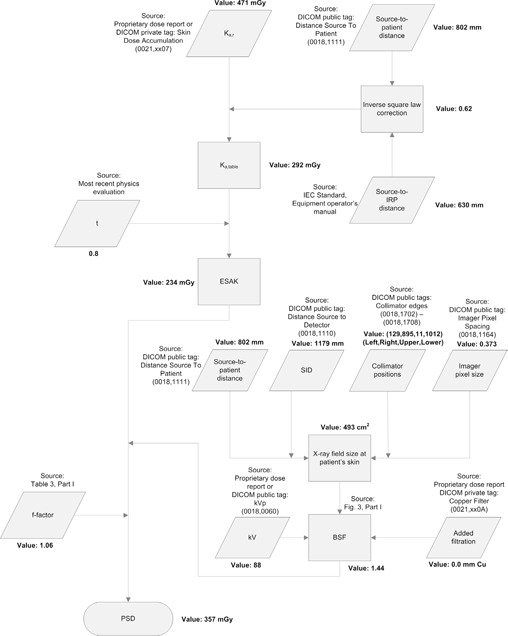
Flowchart outlining the methods and sources of data used to estimate the PSD for a single digital acquisition series. The same method was used to estimate the PSD for other digital acquisition series and fluoroscopy.

The resulting skin doses for each DAS were summed to yield the skin dose exclusive of contributions from fluoroscopic imaging. The $K_{a,r}(f)$ was calculated by subtracting $K_{a,r}(d)$ from the total reference point air kerma, $K_{a,r}(t)$ (Eq. (7), Part I)^(^ ^1^ ^)^. Steps similar to those listed above were followed to estimate the skin dose resulting from fluoroscopy. Fluoroscopic technical factors were estimated based on measured data. The PSD was estimated to be 12.2 Gy. The calculation steps for a single DAS from this case are outlined along the flowchart in Fig. 3. The PSD for this case could easily have been estimated in an aggregate fashion without considering each individual DAS by using only $K_{a,r}(t)$ and the most appropriate single values for the necessary technical factors.

The skin of this patient was examined 24 hours after the procedure, and a telephone follow‐up was scheduled and performed 4 weeks after the procedure. No skin changes were noted. At the time of this work, it has been almost 2 years since the procedure, and no skin changes have been noted.

### B. Case 2

A 60‐year‐old man weighing 102 kg underwent a two‐part embolization as part of selective internal radionuclide microsphere therapy for metastatic colorectal cancer. The initial embolization procedure was complex owing to an unusually high number of extrahepatic vessels requiring occlusion. The $K_{a,r}$ following this procedure was 14,282 mGy. Ten days later, the patient returned for phase 2 of the microsphere treatment, which involved occlusion of additional vessels to isolate hepatic circulation, followed by infusion of the therapeutic agent. The $K_{a,r}$ following the second part of the treatment was 2,153 mGy. A flat‐panel C‐arm fluoroscope was used to perform both procedures. $K_{a,r}$ and KAP were reported, and archived images were available for consultation. This case falls under Scenario 1 from Part I of this review.^(^ ^1^ ^)^

PSD estimation for both phase 1 and phase 2 of the procedure was performed in an aggregate fashion. The peak ESAK was calculated using an inverse square law correction based on measurements of the distance from the focal spot to the underside of the table made by a technologist during the procedure. Attenuation by the table and pad were not accounted for by the $K_{a,r}$ calibration of the fluoroscope, and a standard value from the previous physics evaluation of the unit was used. The peak ESAK values calculated for phase 1 and phase 2 were 8,107 mGy and 1,222 mGy, respectively.

**Figure 4:**
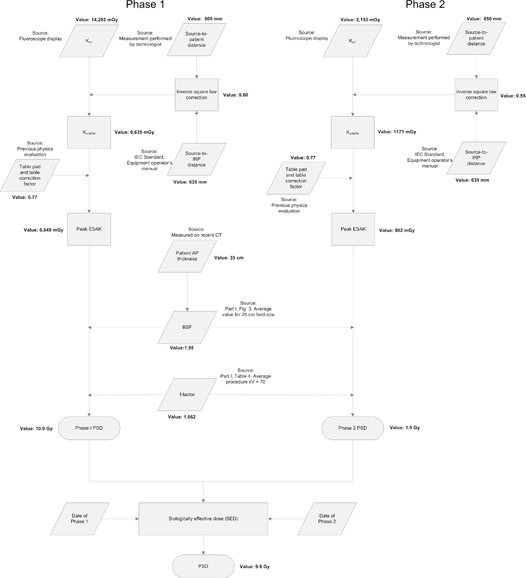
Flowchart outlining the methods and sources of data used to estimate the PSD for Case 2. The concept of the biologically effective dose (BED) is presented in Balter et al.^(^ ^5^ ^)^

To determine the BSF, archived images from the procedure were audited to determine the most used field size. The beam quality (HVL), however, was not readily available. The BSF was estimated based on the most used field size and the patient thickness using Fig. 3 of Part I of this report^(^ ^1^ ^)^ for both fluoroscopic mode and digital acquisition mode. Because calculations were performed in aggregate, the BSFs for fluoroscopic and acquisition modes were averaged before calculating the PSD. In a similar fashion, the f‐factor was determined from Table 4 of Part 1 of this report.^(^ ^1^ ^)^ After application of the BSF and f‐factor, the estimated skin doses were 10,945 mGy and 1,650 mGy, respectively. Analysis of the archived images suggested a maximum workload at a single skin site of 90% owing to use of rotational angiography during both procedures. An adjustment was applied based on this workload, and the calculated PSD values were 9.9 Gy and 1.5 Gy. The calculation steps used in this case are outlined in Fig. 4. This patient experienced a main erythema approximately 3 weeks after phase 1 of the procedure. Commensurate with expectations at this level of dose,^(^ ^5^ ^)^ the erythema was prolonged but cleared 1 month after the initial observation.

### C. Case 3

A 53‐year‐old woman weighing 82 kg underwent a transjugular intrahepatic portosystemic shunt (TIPS) creation procedure. The procedure was lengthy, requiring 50.8 minutes of fluoroscopy time and 471 digital acquisition frames. An XRII C‐arm fluoroscope was used to perform the procedure. The equipment reported only fluoroscopy time and no dosimetric quantities, but archived images were available for consultation. Because no dose metrics were available, the PSD was estimated based on data collected during interviews of staff, analysis of archived images, and measurements performed during a simulation of the case. This case falls under Scenario 2 from Part I of this review.^(^ ^1^ ^)^

According to their training, the technologists involved measured the SPD during the procedure after the fluoroscopy time exceeded 30 minutes, and they also noted that the table height remained constant throughout the case. The physician who originally performed the procedure was interviewed to determine the most‐used operating modes in both fluoroscopy and digital acquisition (e.g., pulse rate, automatic exposure control settings). An estimate of the distance between the anterior surface of the patient and the image receptor was supplied by the physician. This distance, which was used in conjunction with the SPD and patient thickness to determine the SID, is a necessary factor when procedure simulation and measurements are used to estimate the ESAK. A review of archived images was used to determine the FOVs (electronic magnification modes) used for the procedure. In this case, the same FOV was used for approximately 90% of the archived digital acquisition images and was assumed to be used for the same fraction of fluoroscopy.

**Figure 5:**
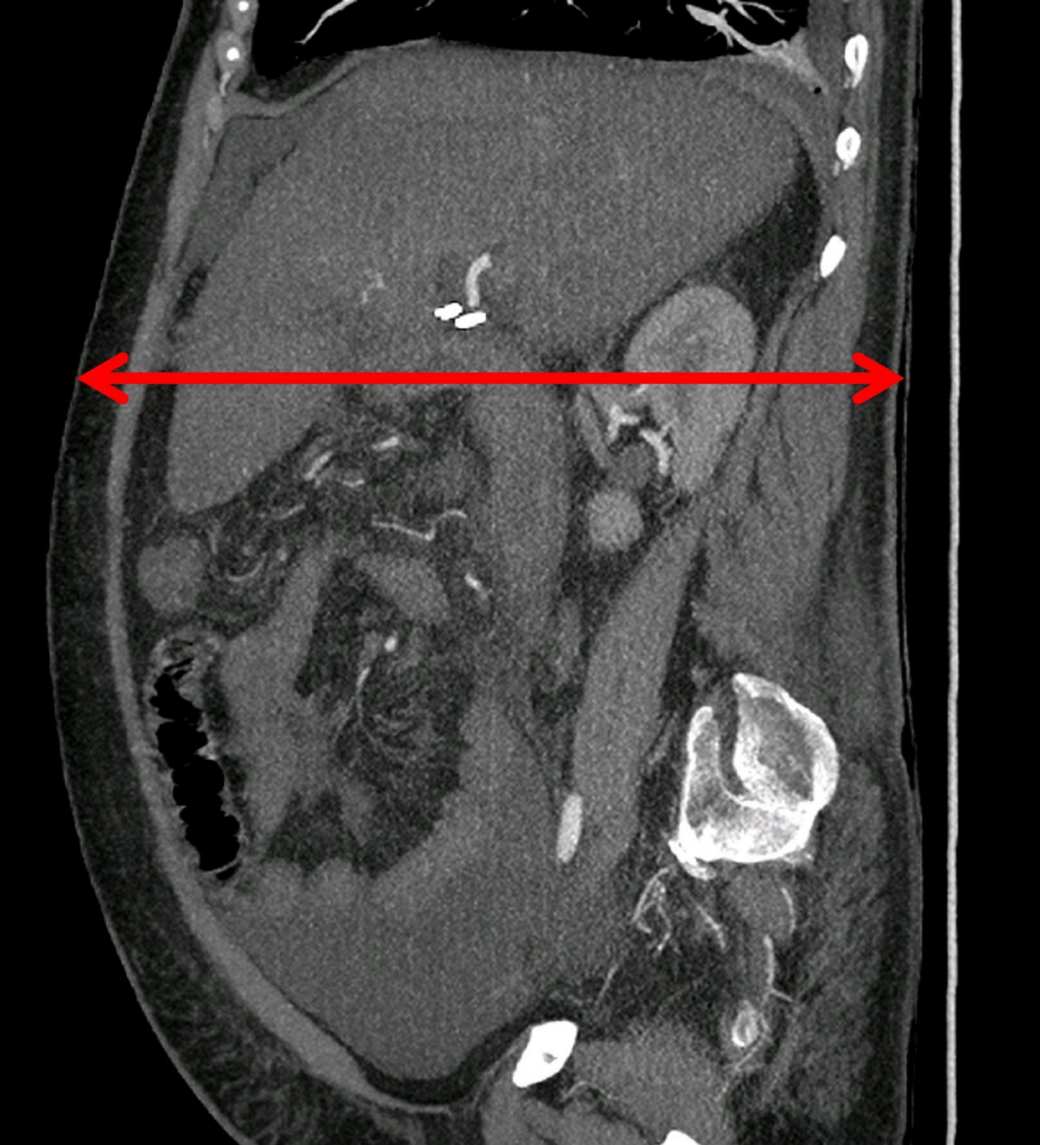
Image from postprocedural CT in Case 3 that was used to measure patient thickness in the anteroposterior direction. Patient thickness was measured at the location of the newly created shunt, which is visible in the image.

**Figure 6:**
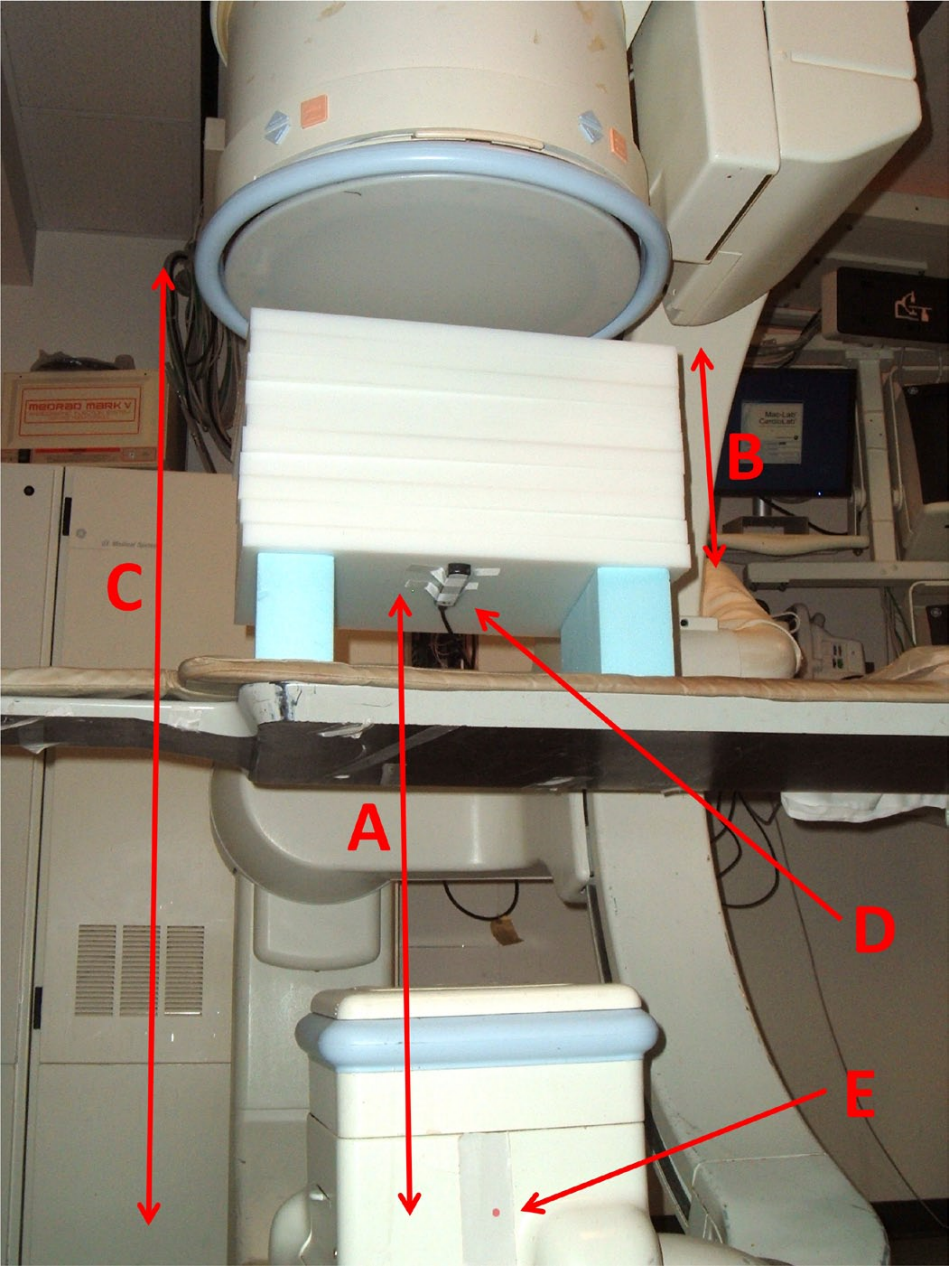
Simulation setup used to measure data for estimation of PSD when no dose metrics were available. The setup was based on recorded procedural information and a review of archived images: (a) source‐to‐chamber distance, which was equal to the SPD used during the actual procedure; (b) patient‐equivalent thickness of polymethylmethacrylate (PMMA); (c) SID as used during the actual procedure; (d) ionization chamber or solid‐state dosimeter; (e) indicated focal spot on X‐ray tube housing.

A suitable patient‐equivalent phantom was used to simulate the procedure for physical measurement of required dose rates. The patient thickness was estimated to be 32 cm based on a measurement obtained from a postprocedure CT scan at the location of the shunt (Fig. 5). The SID was estimated based on the SPD recorded by the technologist, patient thickness, and the distance between the patient and image receptor, which was provided by the physician. Figure 6 shows the experimental setup. Note in Fig. 6(d) that a solid‐state dosimeter was used. While both ionization chambers and solid‐state devices are acceptable for measurement of EAKR, most modern solid‐state dosimeters also report the HVL, which can be used to determine the f‐factors and BSFs for multiple operating modes.

Two measurements of ESAK rate were made during the simulation, one in fluoroscopic imaging mode and one in digital acquisition imaging mode. The ESAK for fluoroscopy was estimated by multiplying the measured ESAK rate by the recorded fluoroscopy time, and estimated for digital acquisition by multiplying the measured dose per frame by the total number of frames.

**Figure 7:**
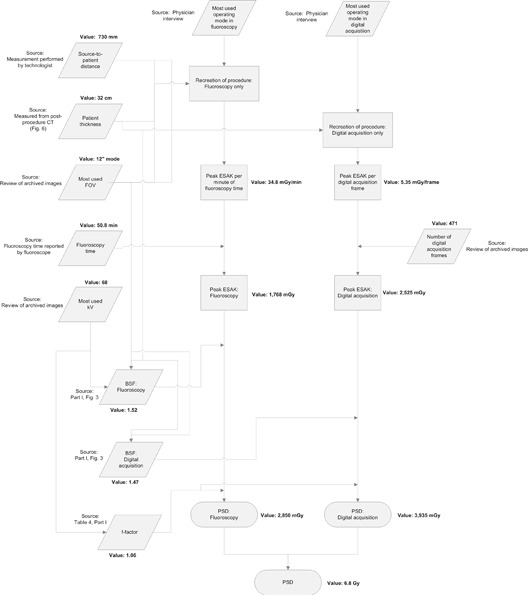
Flowchart outlining the methods and sources of data used to estimate the PSD for Case 3.

The HVL was measured for both fluoroscopic and digital acquisition modes during the simulation of the case. The BSFs for each mode were estimated and applied along with the f‐factors to estimate the PSD. Because no rotational angiography was performed and the X‐ray field size was large and centered over a single skin site, no workload adjustment was made to the PSD. The estimated PSD for this procedure was 6.8 Gy. The calculation steps used in this case are outlined in Fig. 7.

This patient experienced no skin reactions.

### D. Case 4

A 62‐year‐old man weighing 91 kg underwent a complicated coronary angioplasty procedure. No technologist or physician measurements or notes were recorded during the procedure; however, the medical physicist was notified that the total KAP was 440 $\text{Gy}-{\text{cm}}^{2}$. In an interventional radiology case, in which large X‐ray field sizes are commonly used, such a KAP would not be unusual. However, on review of the images, the medical physicist noted that the 6” FOV (electronic magnification mode) was used for greater than 90% of the procedure. Considering the small FOV and large KAP, a detailed PSD reconstruction was deemed necessary to determine the potential for skin reactions in this case. An XRII C‐arm fluoroscope was used to perform the procedure. Fluoroscopy time and KAP were reported by the system, and images were available for consultation. This case falls under Scenario 4 from Part I of this review.^(^ ^1^ ^)^

The calculation was performed in aggregate because no dose reports or DICOM header information were available. The medical physicist met with the technologist and the cardiologist who performed the procedure to obtain an estimate of the SPD and SID. $A_{\text{skin}}$ was calculated using the FOV used for the procedure and the ratio of the SPD to SID.

**Figure 8:**
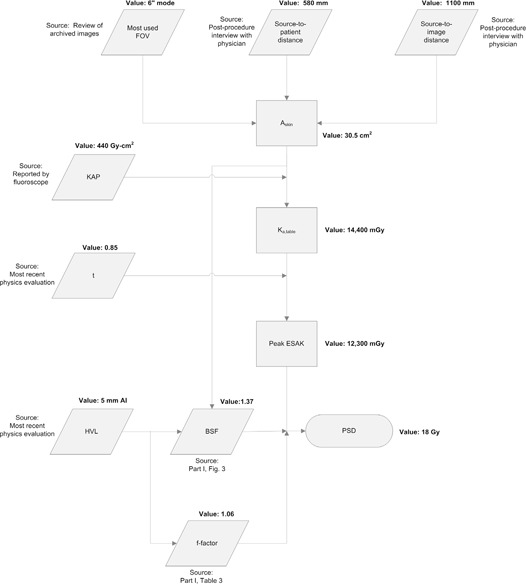
Flowchart outlining the methods and sources of data used to estimate the PSD for Case 4.

The ESAK was estimated by dividing the KAP by $A_{\text{skin}}$ and correcting for table and table pad attenuation. A single measurement of the table and table pad attenuation made during the most recent physics evaluation in fluoroscopic mode was used. The BSF was computed using the HVL measured during the most recent physics evaluation, although it was not necessarily representative of a patient of this thickness. After the BSF and the f‐factor were applied, the PSD was estimated to be 18 Gy. The calculation steps used during this case are outlined in Fig. 8.

This patient experienced mild erythema that quickly cleared within a few weeks of the procedure, an injury that was less severe than expected considering the large estimated PSD.^(^ ^5^ ^)^ This result may be due to rotation of the C‐arm between multiple distinct skin sites, a strategy that may reduce the PSD when using small FOVs^(^ ^6^ ^,^ ^7^ ^)^ but not when using large FOVs.^(^ ^8^ ^)^ Knowing this for certain is impossible, owing to the lack of dose metrics and information available on the fluoroscope used to perform the procedure.

## III. SUMMARY

Estimating the peak skin dose resulting from a fluoroscopically‐guided procedure is a complex task. The complexity and accuracy of the estimate depend on many factors, including the availability of dosimetric quantities reported by the equipment, the availability of other sources of information related to the procedure, and the level of detail desired in the estimation. In the end, the goal is usually to assess the likelihood that the patient will experience deterministic skin changes as a result of the delivered dose.

In light of this goal, and considering that the most recent report on skin reactions stratifies risk into broad dose bands,^(^ ^5^ ^)^ it is in most cases not necessary to achieve accuracy within a few percent when estimating the PSD. Instead, the ability to classify the patient within the appropriate dose band is most important. These bands are 0–2, 2–5, 5–10, 10–15, and >15 Gy^(^ ^5^ ^)^. Also, equipment‐reported dose metrics including $K_{a,r}$ are required to be accurate only to within ±35%.^(^ ^9^ ^)^ This is likely to be the largest source of error when using equipment‐reported dose metrics, and even if all available information is utilized, the accuracy of the PSD estimate may be in error by as much as ±35%, owing to factors that may be outside the control of the medical physicist. However, correction factors can be established for KAP meters that are known to be poorly calibrated. Also, when only KAP is reported by the equipment, large errors may be present in calculated peak skin doses owing to uncertainties in X‐ray field size estimation. This has been discussed at length by other investigators.^(^ ^2^ ^)^ Finally, the accuracy of $K_{a,r}$ and KAP values reported by equipment using a KAP meter varies as a function of beam quality and X‐ray field size.

In some cases there may be uncertainty as to whether or not all imaging that contributed to the peak skin dose has been considered in the dose estimation. While all fluoroscopic contributions can be accounted for by using the recorded fluoroscopy time, some contributions from digital acquisition imaging may be missed if images are acquired but not stored in a permanent archive. This is identical to Scenario 3 presented in Part I of this review,^(^ ^1^ ^)^ and accurate estimation of the PSD may not possible in cases such as this.

Considering the myriad sources of error and their possible magnitudes, simplification of calculations by using a single value for the BSF and f‐factor is justifiable, and in the majority of cases this strategy can be used for the SPD and SID, as well. This simplifies to a consideration of the total $K_{a,r}$ in aggregate, and future work should evaluate the errors introduced when this approach is taken, compared to a piecemeal calculation of the PSD.

In situations where necessary pieces of information are known to be not reported by the imaging system, the medical physicist should tailor his evaluation of the equipment and instruction to the staff to fill in these gaps. For example, if the table height is not reported by the equipment, the medical physicist should instruct the technologist to measure the distance from the source to the patient once a specified $K_{a,r}$ threshold has been reached. NCRP Report 168 has published guidelines on establishing such thresholds.^(^ ^10^ ^)^ The broad beam attenuation of the table and table pad should be measured at acceptance testing if the calibration of the KAP meter does not explicitly account for this factor. Other knowledge gaps can be filled in similarly. Finally, the availability of dosimetric quantities and their reporting should also be considered in the specification of new equipment to be used in the performance of fluoroscopically guided procedures.^(^ ^10^ ^)^
